# Mantram Repetition as a Portable Mindfulness Practice: Applications During the COVID-19 Pandemic

**DOI:** 10.1007/s12671-020-01545-w

**Published:** 2020-11-16

**Authors:** Doug Oman, Jill E. Bormann, Jim J. Kane

**Affiliations:** 1 School of Public Health, University of California, 2121 Berkeley Way, Room #5302, Berkeley, CA 94720-7360 USA; 2 Hahn School of Nursing and Health Sciences/Beyster Institute of Nursing Research, University of San Diego, 5998 Alcala Park, San Diego, CA 92110-2492 USA; 3 Department of Education, Development & Research, Hillcrest Medical Center, University of California, San Diego, 402 Dickinson St. Mail Code 8929, San Diego, CA 92103 USA

**Keywords:** Stress management, Mantra, Spirituality, Diversity, Resilience, COVID-19

## Abstract

**Objective:**

Mantram or holy name repetition has long been practiced in every major religious tradition. Repetition of a mantram as a mindfulness practice is helpful for stress management and resilience building. The objective of this article is to provide an overview of the key features of mantram and the Mantram Repetition Program (MRP) developed in the US Veterans Healthcare System, the evidence base for the MRP, and its applications.

**Methods:**

MRP practices are portable and do not require an extended or regularized period of sitting, in contrast to most methods of meditation. Core functions of MRP practices include focus shifting, frame activation, and fostering of mindfulness. We review scientific research, including multiple randomized trials, that has investigated the MRP.

**Results:**

Research on the MRP has documented reductions in posttraumatic stress symptoms, insomnia, hyperarousal, and depression, as well as enhancement of quality of life, self-efficacy, and mindfulness. Mantram repetition may possess comparative advantages for managing symptoms of various mental health conditions, including posttraumatic stress disorder, HIV/AIDS, cancer, and chronic diseases.

**Conclusions:**

MRP practices can be integrated into daily routines to manage stress and improve coping, safety, efficacy, calmness, and resilience. The inclusion of mantram repetition alongside conventional mindfulness-based practices for stress management will improve their cultural and religious inclusiveness, enabling societies and organizations to build greater collective resilience. Mantram repetition can be readily used in the COVID-19 pandemic, focusing on healthcare workers, patients, and the public.

Frequent mantram repetition is a mindfulness-fostering practice that is often neglected in mindfulness literature, despite the longstanding use of mantram (or mantra) repetition in Buddhism, and the widespread use of mantram practices in the USA. Now a focus of scientific study, mantram repetition possesses a long history as a traditional religious practice: repetition of a single short word, phrase, or prayer—commonly called a mantram in Eastern traditions or a holy name in Western traditions—has been used for centuries in every major religious tradition, often as a portable practice that does *not* require an extended or regularized period of sitting, in contrast to most methods of meditation (Bormann and Oman 2007; Oman 2010, 2020).

Among early Christian fathers, John Cassian (365–435 C.E.) repeatedly prayed Psalm 70:1, advising that “The thought of this verse should be turning unceasingly in your heart... in whatever task or service or journey you find yourself.... as you sleep, as you eat…” (Cassian and Luibhéid 1985, p. 135). Mahatma Gandhi referred to mantram repetition as “Ramanama,” speaking of it as his “staff of life,” and urging those around him to “persevere and ceaselessly repeat Ramanama during all their waking hours,” affirming that “a Christian may find the same solace from the repetition of the name of Jesus, and a Muslim from the name of Allah. All these things have the same implications and they produce identical results under identical circumstances” (Gandhi 1949/2011, p. 5). Tibetan Buddhist leader Dilgo Khyentse Rinpoche taught that repetition of the mantram *Om Mani Padme Hum* “contains the essence of the Dharma” (Rinpoche and Rinpoche 1992, p. 58). And the fourteenth century English author of *The Cloud of Unknowing* advised that one should “Fasten this word to your heart so that it never leaves you, come what may. This word is to be your shield and your spear” (Llewelyn 1983, p. 18). Such portable mantram repetition has been called holy name repetition or monologistic prayer in Christianity, *zikr* in Islam, *japa* in Hinduism, *nembutsu* in Buddhism, and *gerushin* in Judaism (Hausherr 1978; Jones 2001; Oman and Driskill 2003; Schimmel 1975; Shapiro 1997; Tulpule 1991).

## Mantram Repetition Program

As a portable practice used throughout the day, mantram repetition has been empirically investigated mainly through the Mantram Repetition Program (MRP) developed in the US Veterans Administration (VA) Healthcare System. The MRP program instructs each individual to begin by choosing a particular mantram (sacred word or phrase) that he or she will repeat silently on many occasions throughout the day. Participants are encouraged to choose a mantram from a spiritual tradition rather than making up their own word, phrase, affirmation, slogan, or motto. Examples of mantrams supplied in the MRP include *Rama* (used by Mahatma Gandhi), Jesus, *Ave Maria*, *Ribono Shel Olam*, *Allah*, *Namo Amida Butsu*, *Om Mani Padme Hum* (“Jewel [self] in the lotus of the heart”), and My God and My All (used by Francis of Assisi). For additional examples of mantrams as presented in the MRP see Bormann et al. (2020) or Easwaran (2008, 2013). Over the long term, a tradition-derived mantram is viewed as more powerful, stabilizing, and able to provide access to psychological and psycho-spiritual resources (Bormann and Oman 2007).

As taught in the MRP program, guidance in choosing a mantram and integrating its repetition into one’s daily routine has been coupled with two related practices to enhance its effectiveness: slowing down and one-pointed or focused attention. These latter practices, sometimes called “mindfulness points,” work together synergistically to retrain attention, but can also be implemented separately: slowing down to set priorities while limiting activities to avoid chronic time-urgency perceptions; and one-pointed attention to work efficiently while avoiding maladaptive multitasking (Courage et al. 2015; Oman et al. 2008a). As conceptualized in the MRP, these practices were first codified in the late 1960s within a broader nonsectarian system of meditative practices taught by Eknath Easwaran (1910–1999), an Indian-born former literature professor and Fulbright Scholar (Easwaran 2008, 2013; Oman and Bormann 2018).

The MRP itself has sometimes been delivered in as few as 90 min and has been conveyed using a variety of webinar formats (Bormann and Abraham 2019). Most often, the MRP has been offered to VA patients by clinicians in 8 to 16 h over several weekly face-to-face group meetings, or sometimes within individual meetings with patients (Pelletier and Bormann 2018). To prevent confusion, presentations of the MRP typically emphasize that portable mantram repetition and the MRP are structured very differently than mantram-focused methods of sitting meditation (such as Transcendental Meditation, Oman 2020; see also Wachholtz et al. 2017).

### Psychological Functions

Mantram repetition can be understood as serving two overarching psychological functions that are mutually supportive and interconnected: (1) managing immediate stress and (2) building long-term resilience. The first function is primary. It takes place when the mantram is used to render an immediately stressful situation more tractable. The second function, building resilience, complements the stress management function. To build resilience, the mantram can be repeated not only during stressful events, but also during periods—whether days or mere moments—when a person is comparatively free from stress. In the MRP, repetition of a mantram is encouraged during such free moments throughout the day to enable it to be readily mentally available during future stressors. The mantram can also be repeated in response to self-chosen cues, such as when walking down a hallway or before meeting new clients. More broadly, the benefits of repetition are cumulative, “just as an advertiser’s jingle facilitates purchasing an advertised product, constant repetition of a mantram fosters remembrance of [associated] ideals and states of mind” (Bormann and Oman 2007, p. 97). MRP instructional materials, as well as Easwaran’s (2008, 2013) original writings and teachings, encourage training oneself to repeat when walking, when falling asleep, or when waiting in queues. Consistent with research on habits, such repetition is viewed as facilitating the overlearning and hyperstabilization of skills for recalling and benefiting from the mantram, thereby strengthening long-term coping capacities (Arnaudova et al. 2018; Potthoff et al. 2017, 2018; Shibata et al. 2017).

### Research on the MRP

The MRP has been empirically investigated in the USA and Korea in five randomized controlled trials (RCTs) and four mixed-method and three qualitative investigations. In the VA Healthcare System, the MRP has been found useful for stress and symptom management in combat veterans with posttraumatic stress disorder (PTSD), people living with HIV/AIDS and cancer, and veterans with other chronic diseases (Bormann et al. 2006b, 2008, 2013b, 2014b, 2018; Buttner et al. 2016; see also Yong et al. 2018). It has also been found beneficial in healthy populations such as healthcare workers in the USA, nurse managers in Korea, pregnant women, and homeless women (Barger et al. 2015; Bormann et al. 2006a, c, 2017; Hunter et al. 2011; Yong et al. 2011).

#### Mantram Outcomes

Randomized controlled trials (RCTs) of the MRP have documented increases in mindfulness as well as reduced stress and improved coping in veterans with combat-related PTSD and decreased anger in HIV-infected adults (Bormann and Carrico 2009; Bormann et al. 2008, 2014a). RCTs of the MRP have also documented reduced PTSD diagnostic prevalence, reduced PTSD symptom severity, and improved self-efficacy for managing PTSD symptoms (Bormann et al. 2008, 2013b, 2018; Oman and Bormann 2015). RCTs of the MRP have furthermore documented significant and favorable treatment effects on stress-related mental health outcomes that include hyperarousal, insomnia, burnout, quality of life, and depression (Barger et al. 2015; Beck et al. 2017; Bormann et al. 2008, 2013b, 2018; Crawford et al. 2019; Yong et al. 2011). In non-randomized studies, the MRP has been linked to reduced stress in VA staff, family caregivers in the USA, and nursing students in Korea (Bormann et al. 2006c, 2009; Kang and Yong 2019). In addition, MRP practices have fostered increases in overall and existential spiritual well-being, with one reported benefit of mantram practice being enhanced spirituality, and greater mantram use linked to significantly greater gains in spiritual faith/assurance (Bormann et al. 2006a, b, 2013b; Kemppainen et al. 2012; Yong et al. 2011).

#### Mantram in Daily Coping

How people use mantram repetition in daily coping, and peoples’ perceptions and experiences of mantram repetition have been investigated through qualitative interview studies of the MRP. These qualitative studies have focused especially on patients and healthcare workers. Three studies used the critical incident technique, a qualitative method designed to help participants “be as specific as possible in describing specific incidents from memory and to include all relevant details” (Kemppainen 2000, p. 1265). Findings reveal that patients and/or hospital employees have commonly reported using mantram repetition to help manage responses to stress, emotions, physical symptoms and pain, insomnia or other sleep disturbances, unwanted thoughts, flashbacks, and potentially triggering events. They have also used mantram repetition to assist in developing positive qualities such as calmness and/or peace, focused attention, clear thought, enhanced relationships, increased awareness, and increased spirituality (Bormann et al. 2006c, 2013a; Kemppainen et al. 2012).

Qualitative findings also show that repeating a mantram can shift attention away from unhelpful or contra-indicated impulses, thereby supporting goals such as adherence to health behaviors. For example, an MRP participant reported using the mantram “sometimes when I’m on the treadmill at the gym. When I’m wishing that the time would go a little faster. And I’ll just start using my mantram and then I forget about it and it helps me exercise a little longer” (Bormann et al. 2006c, p. 508).

Other examples of MRP experiences reported by patients include using the mantram as an alternative focus for attention to mitigate mortality fears and symptom-related hypervigilance, rumination, distress, and emotional triggers. For example, patients have reported the successful use of mantram repetition to ease breathing during pneumonia (Kemppainen et al. 2012). The mantram has also been used beneficially by HIV patients when fearful of routine medical screening, and by combat veterans with PTSD when unable to sleep due to a nightmare, or when encountering road rage, when triggered by hearing a helicopter overhead, or when dealing with death (Bormann et al. 2013a; Kemppainen et al. 2012). One veteran reported that “the death issues really cause me to repeat [the mantram]. I needed [the mantram] during that period of time to help me” (Bormann et al. 2013a, p. 779). The mantram has also been employed by homeless women living on streets when feeling fear or anxiety (Weinrich et al. 2016).

Among nurses, physicians, and other healthcare workers, semi-structured interviews have been used to elicit not only experiential accounts of mantram repetition but also experiences of overall impact of training on personal and professional lives. In this research, healthcare workers reported multiple ways that mantram repetition, in conjunction with MRP points of slowing down and focused attention, have supported effectiveness at work (Oman et al. 2008b; Richards et al. 2006). Mantram repetition has been repeatedly reported as making it possible to engage in the other core MRP practices, which in turn enabled healthcare workers to “effect changes in their work habits” and “focus their attention on what was at hand” (Oman et al. 2008b, p. 1129). In particular, the mantram was used “in the face of specific difficulties to calm and center one’s self in the course of adversity [and] as a vehicle for slowing down [that provides] a momentary pause to clear the mind, regroup thoughts, and move forward with clearer intuition based on a focused attention… The ability to focus attention on the task at hand without intrusive thoughts or emotions created mental stability in distractive and taxing work environments” (Richards et al. 2006, p. 238). Examples include using the mantram to remain calm when unjustly verbally abused by frustrated families of patients (Richards et al. 2006).

### How the MRP Works

Across disciplines, multiple and perhaps complementary mechanisms have been cited to explain health-related outcomes from repeating a mantram or sacred word or phrase. Theorized or empirically supported mechanisms have ranged from physiological processes to psychological expectancies (Berkovich-Ohana et al. 2015; Bernardi et al. 2001; Qorbani-Vanajemi et al. 2019).

Empirical research offers perspectives on both short-term and longer-term mechanisms through which MRP practices affect outcomes of interest. Regarding short-term mechanisms, fostering mindful awareness may be viewed as one immediate core function of the MRP’s three synergistic practices. Consistent with this possibility, and with other research on mindfulness, mindful awareness has been found in RCTs to mediate MRP benefits that include reduced PTSD symptoms, reduced depression, and increased psychological well-being (Bormann et al. 2014a; Gu et al. 2015; Xia et al. 2019). For example, one patient with PTSD reported that after anguishing memories are triggered, “the mantram… brings me back to touch reality… and I say ‘… I’m here now and I’m okay’” (Bormann et al. 2013a, p. 779). Two additional short-term (proximate) psychological mechanisms are also empirically supported as contributors to effects from the MRP: Focus shifting and frame activation (Bormann et al. 2006b, c; Kemppainen et al. 2012). Focus shifting is documented in numerous reports by MRP participants who employed mantram repetition to shift attention away from loss-of-control feelings such as anxiety, insomnia, or unhelpful thoughts; that is, the mantram has been repeated “during times of emotional turmoil as a means of redirecting attention and interrupting negative ruminative thoughts” (Bormann et al. 2006c, p. 504). Finally, frame activation occurs when mantram repetition activates adaptive, coping-supportive mental frameworks. Compared to meditating on a purely secular word or phrase, RCTs have reported that meditating on a spiritually meaningful mantram produces greater average improvements in anxiety, affect, pain tolerance, reduced migraine frequency, and reduced medication usage (Wachholtz et al. 2017; Wachholtz and Pargament 2008). Such benefits may occur because a spiritually meaningful mantram activates mental perspectives that facilitate viewing life challenges through a “spiritual lens” (Wachholtz and Pargament 2008, p. 363) and facilitating access to salutary forms of religious/spiritual coping with stress (Ano and Vasconcelles 2005; Bormann and Oman 2007; Collins and Loftus 1975; Oman 2018; Pargament 1997).

Over longer time periods of weeks, months, or years, the MRP serves the two complementary overarching functions of managing immediate stress and building long-term resilience. In such longer time periods, the accumulated benefits of engaging in MRP practices and integrating adherence to them into daily routines can foster additional attentional and psychosocial factors that causally mediate outcomes of interest. Thus, statistical mediation analyses of evidence from randomized trials of the MRP, investigating changes over 5 to 8 weeks and sometimes longer, have supported mediation of benefits by at least five factors: increased trait mindfulness, strengthened self-efficacy, improved coping, reduced hyperarousal, and enhanced psychospiritual well-being (Bormann and Carrico 2009; Bormann et al. 2012, 2014a; Crawford et al. 2019; Oman and Bormann 2015). Evidence from RCTs also indicates that gains in mindful awareness from the MRP intervention are mediated by frequency of mantram repetition (Bormann et al. 2014a).

### Mantram, Diversity, and Collective Resilience

Mindfulness is now one of the most commonly recommended stress management approaches, making it a natural fit for collective resilience-building efforts (Joyce et al. 2018). However, the cultural inclusiveness of conventional mindfulness approaches has been contested and is an issue of increasing discussion and concern (e.g., Brown 2017; DeLuca et al. 2018; Palitsky and Kaplan 2019). Arguably needed in the USA and around the world is a richer array of mindfulness approaches that is more culturally inclusive and enhances sensitivity to diversity. No single addition to the conventional mindfulness toolkit seems likely to address all of these concerns. But for several reasons, portable mantram-based approaches arguably represent a natural complement to conventional mindfulness for constructing a more proactively inclusive mindfulness-based stress management toolkit.

First, US national surveys attest to the specific interest in mantram-oriented meditation, practiced by about 1.6% of the US population (about 3.6 million adults), very similar to the 1.9% who practice mindfulness meditation, despite comparatively much less media promotion (Burke et al. 2017). Similarly, among US college students, quasi-experiments have revealed approximately equivalent levels of preference for mantram-based versus mindfulness meditation, refuting suggestions that conventional mindfulness functions as an appropriate “one size fits all” approach (Burke 2012).

Perhaps equally important, as developed in the MRP, mantram-based approaches proactively acknowledge and integrate salutary particularities of diverse major religious traditions (e.g., the mantrams themselves). Such explicit yet non-directive inclusion signals acceptance and facilitates participants’ drawing upon the mindfulness analogues within each tradition—indeed, as affirmed in the inaugural editorial of *Mindfulness*, “mindfulness is ubiquitous in all wisdom traditions… and there is much to learn from these traditions” (Singh 2010, p. 2). Such proactive acceptance-signaling would arguably reduce the likelihood that mindfulness instructors or group participants will inadvertently perpetuate anti-theistic or anti-religious microaggressions by dismissing non-Buddhist religious traditions as inferior or superfluous (Cheng et al. 2019). Moreover, in the USA and in most other countries worldwide, large majorities of people identify as religious and/or spiritual, and such engagement has been consistently linked to better individual health (Chida et al. 2009; Oman 2018; Oman and Syme 2018). Passively allowing cultural or psychological barriers to be erected and normalized between mindfulness and recipients’ preexisting traditions, whether through insufficient proactivity or unintentional micro-aggressions, risks “the trap of adopting a purely etic (outsider) perspective,” while remaining ironically unmindful of “emic” (insider) perspectives (Walsh and Shapiro 2006, p. 228).

Such considerations argue that community planners and policy makers should foster improved cultural balance in supported mindfulness programs. One possible starting point for such rebalancing could be to recognize and when feasible proactively include mantram repetition training alongside conventional mindfulness or other stress management modalities (Oman and Bormann 2020). Such expanded toolkits could be used for purposes ranging from workplace stress reduction to pandemic resilience preparation. This has been done, for example, in the VA Healthcare System, where the MRP has been offered alongside other approaches as a primary intervention for symptom and stress management and also as an adjunctive treatment for PTSD (Bormann et al. 2013b, 2018; Buttner et al. 2016). Of course, the optimal balance between portable mantram-based mindfulness, conventional mindfulness, and other modalities may vary between settings, populations, and needs (Bowman 2013; Kuo 2011). Yet diversity considerations suggest that a flexible toolkit that makes available both conventional approaches and mantram will most fully strengthen collective resilience, outperforming exclusive reliance on any single pre-determined approach.

### Expanded Recognition of Implicit Mindfulness

As embodied in the MRP, mantram repetition shows both similarities and differences to other approaches for cultivating mindfulness, even as diverse understandings of mindfulness (*sati*) are evident in different traditions of Buddhism (Theravada, Mahayana, Vajrayana), and in different approaches to mindfulness in modern psychology (e.g., MBSR, MBCT, DBT) (Feng et al. 2018; Rosch 2015). On the one hand, the MRP does not systematically employ the vocabulary and concepts of mindfulness, nor does it assert a greater inheritance from Buddhism than from psychologies of other spiritual wisdom traditions (e.g., Plante 2010; Rao and Paranjpe 2016). As such, portable mantram repetition might be understood as a prototypical form of implicit mindfulness—a practice that fosters mindfulness without invoking mindfulness vocabulary or Buddhist concepts (Xia et al. 2019). But on the other hand, the MRP does possess many commonalities with other approaches that explicitly or implicitly foster mindfulness (Oman and Bormann 2020).

One fundamental commonality is that the MRP teaches people to regulate attention in skillful ways that are consistent with traditional conceptions of mindfulness, which in traditional Buddhism may involve either expanded or focused attention as appropriate to the purpose at hand (Feng et al. 2018). For example, the MRP’s inclusion of the practice of focused or one-pointed attention resonates with traditional teachings that a needed basis for mindfulness (*sati*) is adequate concentration, which can be developed through concentrative practices (e.g., *shamatha*, Wallace 2006). The MRP’s encouragement of focused or one pointed attention also resonates with instructions in some psychological mindfulness approaches to “participate in the moment… one-mindfully” (Linehan and Chen 2005, p. 168). Conversely, the MRP’s core practice of slowing down is understood to motivate and facilitate awareness of all phenomena relevant to effective and purposeful daily living, thereby facilitating awareness of both interoceptive and exteroceptive phenomena (Fox et al. 2014).

An additional commonality is that the MRP’s endorsement of tradition-derived mantrams, understood as supporting connection to the corresponding spiritual ideals and values, is parallel to traditional Buddhist emphases not merely on mindfulness but on *right* mindfulness (*sammā-sati*), which “contains the elements of ethics and wisdom” (Feng et al. 2018, p. 455). Right mindfulness also implies appropriate forms of past awareness, such as recollecting “what was done and said long ago” (*Saṃyutta Nikāya* 48:9), thus “in effect reminding us of who we are and what our values are” (Gethin 2011, p. 270; Oman 2020).

## Applications of MRP

The portability of mantram repetition gives it potential for integration into almost every person’s daily routines, as reflected by the breadth of groups in which the MRP has been studied, ranging from healthcare workers to homeless populations. Methods for training MRP instructors have been developed as part of ongoing research work at the VA (e.g., Buttner et al. 2016). Importantly, beginners typically learn the three MRP practices most effectively when its presentation to them is tailored to their own individual, cultural, social, and material settings and needs. In particular, mantram repetition can be learned most easily when people hear examples of how to utilize the mantram that are relevant to their own daily personal and work environments. Beginners can benefit, for example, by learning examples of how to use the mantram for managing stressors common in their workplaces; learning examples of neutral cues to use for remembering the mantram in non-stressful times; and learning anecdotal accounts that link such mantram utilization with experienced results (see Bormann et al. 2020).

### Application of MRP during the COVID-19 Pandemic

Around the world in 2020, daily life has been transformed by the expanding coronavirus disease pandemic, which is posing new challenges for health providers, patients, families, health systems, and ordinary citizens. Daily news reports from many countries have made it abundantly clear that the COVID-19 pandemic creates highly distressing and sometimes life-threatening challenges for healthcare workers, patients, patients’ families, and the general public (e.g., Shanafelt et al. 2020; Wang et al. 2020a, b; Zhang et al. 2020). These coping challenges require both technical and psychosocial responses. Urgent on the technical side is providing lifesaving treatment and diagnostic testing, building emergency health system capacity, and implementing curve-bending preventive measures such as social distancing. These technical efforts should be accompanied by mental health supports to help sustain efforts by health-care workers and first responders, heal patients, foster social cohesion, and support better decision-making by individuals, organizations, and communities. No single mental health or stress management approach can be optimal for everyone. But based on evidence and experience, portable mantram repetition holds promise to benefit all major groups affected by the pandemic. The following paragraphs illustrate how the mantram may be used by three key groups: healthcare workers, patients, and the general population.

Healthcare workers working with COVID-19 patients, for example, must adhere to life-saving hygiene procedures. Such adherence demands heightened presence of mind and *one-pointed attention* for safe donning and doffing in proper sequence to avoid contamination. In particular, doffing contaminated equipment requires handwashing several times (Centers for Disease Control and Prevention (CDC), 2020) and careful removal of potentially life-threatening equipment such as contaminated goggles, hoods, and gloves. Such doffing must often be done rapidly in order to respond to other emergent situations. Prior *repetition of the mantram* can be used to support the focus needed to adhere to these and other mandated procedures (Bormann et al. 2006c).

Similarly, in COVID-19 “hot spots,” some healthcare workers face the emotionally wrenching challenge of delivering care to individuals who are their close friends and were colleagues only days prior. In such circumstances the MRP’s portable practices can be used to calm emotions while maintaining focus, mindfulness, and compassion. COVID-19 patient care may also demand emotionally draining conversations with patients’ families about end of life issues. Such conversations require focus, clarity, and sensitivity to emotions and to a family’s practical and spiritual concerns. These qualities are supported by MRP practices, which can also facilitate dealing with the healthcare worker’s own emotional sequelae from such conversations. The mantram also can be used to steady the mind when dealing with deaths, rationing, or other existentially challenging issues Table 1 offers numerous examples of stressor and self-cueing occasions that could be mentioned in an MRP training for health professionals engaged in COVID-19-related patient care.

**Table 1:** Examples of healthcare workplace situations usable as cues for mantram repetition

External cue reminder to begin repeating one’s mantram	Purpose/result of repeating mantram
When tackling a routine yet unpleasant or difficult task	To pause, to center oneself, to clear mind of any judgment, fear, distaste
While waiting for anyone or anything soon to arrive	To allow a moment of respite
Walking down the hall	To allow a short respite from external chaos
Handwashing	To strengthen mental concentration in support of essential hygiene; to “own these 20 seconds”
Before donning personal protective equipment (PPE)	To support one-pointed focus, and clear mind for next task
Before doffing PPE	To refocus attention, attend carefully to critical safety procedures, and transition to next task
Upon opening a door when grasping any doorknob or handle	To pause, and to clear and re-center one’s mind
Prior to making eye contact when greeting a new patient	To allow feeling a calm presence prior to conversations
Before speaking with a patient’s family or friends	To clear mind, re-center, remain calm and present
During a challenging team interaction	To maintain calm, and ensure listening and engaging productively; to sustain relationships for future
Prior to having to enforce uncomfortable rules	To help sustain composure, listening, and focusing on the important priority of the regulation; to assist in avoiding unnecessary conflict
When providing healthcare or other caring to a fellow colleague	To clear mind, re-center, remain calm and present
When calling or preparing for an emergency procedure such as resuscitation [Code Blue]	To keep calm, perform accurately, stay mindful
When further treatment is futile and a healthcare worker is the sole attendee at a patient’s dying	To be fully present and support one’s personal resilience; to cope with one’s impotence to prevent death

For patients, COVID-19 also presents distinctive challenges for which MRP practices may be helpful. Respiratory distress is one COVID-19 presenting symptom that is common in more severe cases and can produce hypervigilance to bodily changes (Harrison et al. 2014; Ji et al. 2020; Sohrabi et al. 2020). Patients with dyspnea have reported that “I could not take my mind off breathing for fear my life would end” (Harrison et al. 2014, p. 39), and severe cases may cause PTSD symptoms that persist for many months (Davydow et al. 2008). Conventional mindfulness interventions generally use breathing as a focus of concentration, and evidence to date has not supported the efficacy of such interventions for reducing stress in patients with respiratory illnesses (Harrison et al. 2016; see also Clari et al. 2020). Such conventional mindfulness approaches may be ineffective or even contra-indicated for patients with respiratory distress, as “drawing attention to breathing in those with dyspnea [shortness of breath] could provoke hyper-vigilance of breathless symptoms resulting in emotional distress” (Harrison et al. 2016, p. 349). Instead, such patients may be taught use the mantram to redirect attention away from experiences of shortness of breath.

Similarly, patients with suspected or confirmed COVID-19 illness may use the mantram to manage mortality fears, and if necessary, the potential additional anxiety of undergoing a life-threatening illness when medical care systems could become overwhelmed and unavailable. MRP-fostered gains in spiritual well-being suggest that mantram repetition can help many people to manage illness-related death anxiety, as affirmed in many religious traditions, perhaps in part by mentally activating spiritual perspectives on mortality and longevity (Easwaran 2008; Grossman et al. 2018; Jong et al. 2018; Pargament 1997; Wachholtz and Pargament 2008). Family members who are supporting a patient may benefit by repeating a mantram themselves, and can also repeat a patient’s mantram with them, either aloud (when appropriate) or silently. Corresponding stress reductions may contribute to healing and immunity (Schakel et al. 2019; Taylor 2019).

Finally, in the general population, mantram repetition can support many people in adhering to mandated social distancing and hygienic practices. Mantram repetition can calm the mind and mentally activate psychospiritual frameworks that discount immediate frustration in favor of longer-term benefits and acceptance-based responding (Bormann and Carrico 2009; Carrico et al. 2007). The mantram can also help manage other widespread fears—fears that may sometimes possess validity. Besides the virus itself, people may legitimately fear scapegoating, loss of employment or other economic harm, scarcity of personal protective equipment, separation from family during quarantines and lockdowns, and the sequelae of all these events (Taylor 2019). By one month after the initial outbreak, a significant fraction of the Chinese population was showing posttraumatic stress symptoms (Boyraz and Legros 2020; Sun et al. 2020). By preventing counter-productive rumination about such fears, and by mentally activating spiritual perspectives, the mantram can help mitigate or control the related suffering. Mantram repetition can also be used for comfort by those who are physically separated from family members, whereas families living in close quarters for extended periods may find the mantram an aid to maintaining patience. Small children can be given a mantram to repeat during times of fear or frustration, and it can be sung to them as a lullaby. Entire families can even sing mantrams together.

Unfortunately, vaccine development timelines for COVID-19 are uncertain and may require years until availability is widespread (Arnold 2020; Bregu et al. 2011). Thus, the worldwide yet uneven pursuit of social distancing and other pandemic control efforts raises the possibility that many countries will experience multiple pandemic waves, akin to the 1918–1919 influenza pandemic (and many others, Miller et al. 2009). Indeed, a modeling report from Imperial College has warned that “the more successful a strategy is at temporary suppression, the larger the later epidemic is predicted to be in the absence of vaccination, due to lesser build-up of herd immunity” (Ferguson et al. 2020, p. 11). Irregularities and uncertainties in disease spread may in turn generate additional cascading economic, social, and cultural uncertainties, irregularities, and shocks. And evidence now suggests that COVID-19 has long-lasting effects for a significant fraction of those infected: a study of 143 COVID-19 patients discharged from hospital in Italy found nearly nine in 10 (87%) still experienced at least one symptom after 60 days, and shortness of breath was still reported by nearly half (dyspnea, 43%, Carfi et al.^2020^; see also Mahase 2020). To prepare for multiple pandemic waves, public health and disaster preparedness principles indicate that societies should build both technical and mental health facets of resilience (Aiello et al. 2011; Pfefferbaum et al. 2012)—and there is reason to believe the mantram can play a useful role in preparing.

## Future Directions

Mantram repetition represents an empirically supported and plausible option for many current adjunctive treatment needs and is a readily learned practice by individuals for self-care and self-management (see Bormann et al. 2020). But the widest and most discerning use and dissemination of mantram repetition will require additional knowledge. Foundational scholarship should investigate, for example, how the relation between mantram and mindfulness or its analogues has been theorized within various Buddhist or non-Buddhist traditions (e.g., Pure Land Buddhism, Jones 2001; Hinduism, Maharaj 2013; Christianity, Rehg 2002; and even Theravada Buddhism, Crosby 2000). A variety of practical questions should also be explored, such as the following: In what ways do USA- and Korean-based findings about training and utilization of mantram repetition generalize to workers and populations elsewhere? To what degree does mantram repetition enhance safety, immune competence, and healing? Can mantram repetition help address public health burdens such as the “growing but neglected global epidemic” of chronic obstructive pulmonary disease (with 11.7% estimated global prevalence for ages 30-plus, Adeloye et al. 2015, p. 2; see also Clari et al. 2020)? What materials and procedures are optimal for integrating mantram repetition as a component of stress management toolkits for diverse populations? How can mantram repetition approaches be optimally tailored for different populations worldwide? And on an immediate practical level, are there ways that already available portable mantram repetition resources can enable individuals and organizations to prepare for ongoing waves of the COVID-19 pandemic, or other future collective stressors?

This article has reviewed empirical, historical, and cross-cultural evidence that we believe amply demonstrates the relevance of mantram repetition to the fields of mindfulness, mental health, and stress management, as well as to concerns in those fields for cultural inclusiveness. Further investigations of mantram repetition as a viable and effective aid to lessening suffering and fostering human well-being and thriving are needed.
